# Professor Oleh Hornykiewicz, MD (1926–2020): Remembering the Father of the Modern Treatment of Parkinson's Disease and the Man

**DOI:** 10.1002/mds.28317

**Published:** 2020-09-28

**Authors:** Ali H. Rajput, Stephen J. Kish

**Affiliations:** ^1^ Saskatchewan Movement Disorders Program University of Saskatchewan/Saskatchewan Health Region Saskatoon Saskatchewan Canada; ^2^ Human Brain Laboratory Centre for Addiction and Mental Health Toronto Ontario Canada

**Keywords:** frozen human brain; dopamine; levodopa; Parkinson's disease; Nobel prize

This tribute provides thoughts and recollections by Saskatoon neurologist Ali Rajput and Toronto scientist Stephen Kish on the life of Professor Oleh Hornykiewicz, who passed away on May 26, 2020, in Vienna at the age of 93.

## 1 Early Years and Education

Oleh Hornykiewicz was born in Sychow (Lvov), Ukraine (formerly Poland). When he was 13 years old, his family migrated to Vienna, Austria, during the Second World War. All of his future education was in Vienna. He graduated with a doctorate in medicine from the University of Vienna in 1951.^1^ During undergraduate medical school, he was highly impressed with his professors of organic chemistry, neuroanatomy, and pharmacology. He decided to pursue research that combined those disciplines rather than train for clinical practice.

## 2 First Research Project

After graduation, Oleh joined the Pharmacological Institute at the University of Vienna as an unpaid research assistant to work with Professor Franz von Brucke.^1^ The institute attracted many leading European experts, including Professor Hermann Blaschko of Oxford University, England. Oleh's first research project was the study of ceruloplasmin in Wilson disease. Because Wilson disease involved the basal ganglia, he developed a special interest in their functions.^1^

## 3 A Focus on Dopamine Research

In 1956, he was awarded a British Council Scholarship to work with Professor Blaschko at Oxford University. Dopamine (DA) had been discovered recently but was generally regarded as an intermediate step in the synthesis of adrenaline with no physiological function of its own. Blaschko, on the other hand, believed that DA had some independent regulatory function and directed Oleh to work on DA.^1^ His research project was the study of DA's effect on blood pressure in the guinea pig. He confirmed a previous report that, contrary to the action of adrenaline, which elevated blood pressure, DA lowered blood pressure. He and his supervisor were now convinced that DA played a distinct but unknown physiological role. While at Oxford, he also determined that levodopa (l‐dopa) behaved very much like DA.^1^ When he finished his studies 18 months later, Professor Blaschko advised him to continue working on DA.

## 4 The Role of DA in Human Brain and Parkinson's Disease (PD)

A major goal of medical research is to carry out studies with possible application to human health and disease. Hence, laboratory/animal studies must pass through the phase of validation in human subjects before application to medical teaching and practice. Even today, there is no perfect animal model of PD. The experts in the 1950s and 1960s had serious reservations about biochemical studies of autopsied human brains, such as fresh frozen material, and raised doubt that it would yield any meaningful information, such as pertaining to neurotransmitters.^1^

By the time Oleh returned to Vienna in 1958, he was well versed in laboratory methodology and animal studies, and he had a sound knowledge of biochemistry, pharmacology, and human brain anatomy. He also had an understanding of some physiological functions of DA and l‐dopa. Having confirmed the peripheral physiological effect of DA, he decided to study the role of DA using autopsied human brains. He was now an independent investigator and had postdoctoral students working with him. With his knowledge of neuroanatomy and the conviction that brain tissue must be well suited for laboratory studies of neurological disorders, he devised a method to dissect tissue of research interest from frozen brain for biochemical analysis.

By early 1959, he and his research student, Herbert Ehringer, started systematically analyzing specimens of frozen human brains for their DA and noradrenaline content. With remarkable efficiency, they studied 31 brains in 1 year.^1^ These included brains from neurologically normal human adults, fetuses, parkinsonism cases, and other degenerative disorders such as Huntington's disease. They detected a marked reduction of DA in the striatum of persons with PD and postencephalitic parkinsonism. Their article was published in German^2^ and later translated into English.^3^ For the first time, they demonstrated that freshly frozen postmortem brain could be studied for reliable assessment of neurotransmitter levels. They also demonstrated that striatal DA deficiency was specific for the neurological condition of parkinsonism.

## 5 DA Replacement Experiment

Oleh's next undertaking was to explore whether brain DA deficiency in PD could be corrected pharmacologically. He already knew that DA did not cross the blood–brain barrier, but its precursor l‐dopa did, and the 2 substances had similar physiological effects. In collaboration with a clinical neurologist, Professor Walter Birkmayer, he performed an elegant clinical neuropharmacological experiment. Oleh had access to only 2 g of pure l‐dopa. To determine its efficacy in as many patients with parkinsonian as possible, they decided to use levodopa intravenously. They administered the drug to 20 patients using different dosages: 50, 100, and 150 mg. The patients' responses were “spectacular,” as he would often describe them. The bedridden patients could stand up, and those who previously could not walk could now do so.^4^ The weakest response was on 50 mg, and the most pronounced was with 150 mg.^4^ That article was also published in German in 1961^4^ and later translated in English.^5^ To document their clinical observations, Oleh and colleagues made movies of untreated and post‐l‐dopa‐treated patients; he loaned those movies to experts upon request. The observation that l‐dopa benefit was dose dependent was subsequently incorporated by Cotzias and colleagues into their clinical trials.^6^ They reported dramatic improvements in patients with PD on large oral doses of D‐L‐DOPA.^6^

In the remarkably short span of 2 years, Oleh had established that (1) striatal DA deficiency was a major biochemical abnormality in PD and (2) l‐dopa corrected that deficiency, thereby resulting in major symptomatic benefit. Both were new observations at the time. With those 2 studies, Oleh revolutionized the approach to chronic neurological disease research and laid the foundation of science‐based treatment of the disease. Until then, it was believed that neurodegenerative disorders were untreatable.^1^ Hornykiewicz's model was later adopted for other neurodegenerative diseases such as Alzheimer's disease.^1^ He was also the first scientist to speculate that a pathway from the anterior midbrain to the basal ganglia in the forebrain would underlie the DA content of the striatum in normal human brain.

Oleh was young—in his early 30s—when he made those groundbreaking observations. Many senior scientists were skeptical of his findings. His first 2 articles were published in German in a journal to which few international scientists paid attention. Thus, the English‐speaking world could not fully appreciate the importance of his findings. Nevertheless, the original observations of Oleh Hornykiewicz have stood the test of time. In essentially 60 years, there has never been a publication contradicting either of those 2 studies. Many clinicians and basic scientists made pilgrimages to Professor Hornykiewicz's laboratory for firsthand visits of his set up and to see the movies that he and Birkmayer had made of the treated patients. The acceptance of his work was slow but steady. His future work consolidated the validity of the earlier studies.

## 6 Some Related Questions Resolved

Oleh and his colleagues subsequently established that the severity of striatal DA loss correlated with the severity of substantia nigra neuronal loss and, in turn, with the severity of parkinsonism.^1^, ^7^ He continued studies of human brain and 1‐methyl‐4‐phenyl‐1,2,3,6‐tetrahydropyridine—treated monkey models with support from his very capable junior colleagues: Drs. Ken Lloyd (Toronto), Stephen Kish (Toronto), and Christian Pifl (Vienna).^1^ Oleh also established that brain enzyme dihydroxyphenylalanine (DOPA) decarboxylase converted l‐dopa to DA.^1^ He demonstrated that DA deficiency in PD was specific and not simply a result of normal aging.^8^, ^9^ He studied the biochemical basis of l‐dopa–induced dyskinesia and wearing‐off phenomena.^10^, ^11^ He advanced the idea that PD could be a vesicular storage disorder.^12^

## 7 Second Institution and Second Citizenship

In 1967, Oleh moved to Clarke Institute at the University of Toronto in Ontario, Canada, as a neuropsychopharmacologist to head a newly formed research institute.^1^ He stayed in Toronto for the next 10 years. He also became a dual citizen of Canada and Austria.

## 8 Recollections of the Toronto Years by Dr. Stephen Kish

Professor Oleh Hornykiewicz was my teacher, constant mentor, and scientific colleague. Our association spanned the period from 1979 to 2020, the year of his death. I was based in Toronto, originally in his laboratory at the Clarke Institute of Psychiatry.

For part of his career (1967–1977), Oleh headed the psychopharmacology section at Toronto's Clarke Institute of Psychiatry (now the Centre for Addiction and Mental Health). In 1977, he returned to the University of Vienna and began splitting his time between Vienna and Toronto, where he continued to supervise the Clarke's Human Brain Laboratory until 1992.

Oleh mentioned in the late 1960s (some 10 years following his initial discoveries) that some scientists still expressed skepticism regarding the usefulness of l‐dopa in PD. For amusement purposes, he kept a *Medical Post* article on a debate hosted by the Canadian Royal College of Physicians and Surgeons between a researcher and a neurologist on the benefit of l‐dopa in PD. The researcher expressed “skepticism” because l‐dopa in her animal model failed to show an increase in brain DA levels, whereas the neurologist opined that “proving the drug's efficacy is no longer an issue” and that “the results he has had with l‐dopa are so outstanding that it holds the promise of being the most important advance in the treatment of parkinsonism.”

Hornykiewicz's article of brain DA deficiency in PD was, by today's standards, a “preliminary” finding.^2^ Likely, the first independent replication study was conducted 11 years later in 1971 by Dr. Stanley Fahn.^13^

In Toronto, working with Ken Lloyd, Oleh largely disposed of the question of the role of DA in the therapeutic effect by l‐dopa by showing in a logistically challenging postmortem brain study that brain levels of DA in patients with PD who had received l‐dopa were 0 to 15 times higher than those who had not received such treatment and that the levels were higher in the “good” versus “poor” clinical responders.^14^

## 9 Toronto Studies From 1980 to the Present

I first encountered Oleh in 1979 as a graduate student in the Pharmacology Department at the University of British Columbia in Vancouver. My doctorate thesis was focused (unusually at the time) entirely on biochemical studies of autopsied human brain, and my supervisor agreed to send me to Toronto for a week in 1979 to learn from the master himself, Professor Hornykiewicz, how to carry out the dissection procedure.

I found Hornykiewicz to be kind, supportive, and very much a gentleman, but with his conservative dress (including on weekends) and his unusual, double lens‐carrying, flip‐one‐up spectacles, he represented a rather imposing Austrian professor. He was my constant scientific colleague from 1980 until his death in 2020.

Among the Toronto studies that Oleh considered especially important was the demonstration of the highly specific, subregional pattern of DA loss in the striatum of patients with idiopathic PD^9^ compared with those patterns observed in persons with other parkinsonian conditions. He also discovered that the topographic pattern of DA loss in normal aging was distinct from that in idiopathic PD.^8^ Accordingly, he felt that these findings suggested that the cause of degeneration of DA‐producing neurons in idiopathic PD is likely not the same as that responsible for loss in other parkinsonian conditions (or in normal aging). Along these lines, he would insist that in our joint manuscripts we clarify explicitly that the parkinsonian disorder we studied is idiopathic PD, which is likely to have a specific cause. Oleh also argued for the remainder of his career that studies of hereditary parkinsonism might not be helpful in understanding the cause of idiopathic PD.

However, as a colleague, Oleh was a stickler in joint publications to ensure that we cite the very first person who made a finding “ …because this is how it should be done.” We both enjoyed going back to the very early literature from the 1930s and 1940s (often in German) to discover the now forgotten fellow who was the first person to describe a key finding. Oleh's recollection of scientific facts and research discoveries was astounding right up to the end. The conversations that I had with him in 2020 were at the same level as those 40 years earlier.

## 10 How Will Professor Hornykiewicz Be Remembered?

Oleh made a seminal discovery,^2^ and in the space of less than 2 years translated that pivotal finding into the clinic.^4^ In so doing, he became the founding father of a new era in neuroscience. In the 40 years that I knew Oleh Hornykiewicz, we discussed only once how he would like to be remembered. During this exchange, he mentioned that, if I were to write an obituary of him, I should consider the statement by the much admired British neurologist and scientist, Dr. C. David Marsden. David had summed it up simply and accurately, namely, that for a person with PD, there was a before and after Oleh Hornykiewicz.^15^ Marsden said that his “discovery… changed everything.”

## 11 In Toronto, “What Made Hornykiewicz Tick”?

What made Oleh “really excited” I feel, more than anything else, was in discovering “new regions” of the brain, or better, old regions with new functions, and trying to figure out whether they might be involved in PD. An example was the small, noradrenaline‐rich nucleus accumbens,^16^ which Hornykiewicz subdivided even more, and which only he could reliably dissect from frozen autopsied human brain using his special standardized dissection procedure.

## 12 …and Then There Was the Claustrum

At the age of 90, he spearheaded a study, entirely his own and with little moral support from his junior colleagues, on the behavior of claustrum, with its still uncertain functions, in PD. Sometimes Oleh jokingly pointed to the claustrum during a brain dissection and suggested that the soul of man must be located in that region. Hornykiewicz published the article on the claustrum in 2017 at the age of 90.^17^

## 13 Back to Vienna and a Dual Role

In 1977, he returned to Vienna as Head of the Institute of Biochemical Pharmacology and was appointed Professor of Biochemical Pharmacology.^1^ Henceforth, he split his time between the University of Vienna and the University of Toronto until his mandatory retirement in the 1990s at both institutions. However, his scientific productivity was far from over. His tenure at the helm of neuroscientific research activities in Vienna has recently been summarized by others.^18^

## 14 Recollections of Saskatoon Years by Dr. Ali Rajput

### 14.1 Third Institutional Affiliation

In 1996, Oleh Hornykiewicz was appointed Distinguished Professor of Brain Disorder Research at the University of Saskatchewan in Saskatoon, Canada, a position he retained for the rest of his life.

He was my colleague and friend from 1975 to 2020. In late 1968, we started a movement disorders program in Saskatoon. We conducted longitudinal patient follow‐ups with detailed clinical documentation of each subject that included videography in most cases. By 1970, we started performing autopsies on the deceased patients and soon decided to use a half of the brain for standard pathology and preserve the other half at −80°C for future research.^19^ By the mid‐1970s, Oleh and I had established a collaboration that continued for the rest of his life. Our first article together was published in the journal *Nature* in 1978.^20^

After he left Toronto in 1992, the intensity of our collaboration declined. At a meeting in 1996, I asked Oleh, “Where should we send someone for training to study our postmortem frozen human brains?” Without hesitation he asked, “Do you want me to come to Saskatoon?”, to which he added, “You do not have to pay me.” We could not have wished for a more qualified scientist and at that price! He came to Saskatoon for approximately 1 week at a time, once or twice a year. He had a private office and his own laboratory. We would discuss manuscripts and plan future research, and he would dissect frozen half‐brain samples that were sent to his laboratory in Vienna or to other researchers (Drs. Paul Bedard, Therese Di Paolo, and Frederic Calon) for further studies.^11^ The critical link between my clinical operations and his laboratory work was access to suitable frozen brain samples for analysis. He trained Dr. Alex Rajput who is now the Director of the Saskatchewan Movement Disorders Program in brain dissection here in Saskatoon, ensuring future research.

Oleh was a gentleman and a scholar in the best sense of those words. He was shy, unselfish, and did not promote himself. He had very high professional ethical standards. When we discussed manuscripts, Oleh would go out of his way to give credit to individuals who had done some related work. At times, I would ask him for a reference and he would say, “I heard him say that.” He gave credit without consideration of reciprocity. He was a meticulous writer; he checked every word and every sentence in the manuscript for accuracy. In his autobiographic piece, Oleh noted his association with me: “Without his unique brain material we would not have been able to do even one‐tenth of our human brain research.”^1^ As important as it seems, he turned down my initial proposal to collaborate for ethical reasons. He was told (inaccurately) that I was collaborating with someone else.

During his visits to Saskatoon, my wife would arrange dinners at our house, where Oleh would relax and meet new friends. Coincidentally, my wife's grandfather was born in the same area of Ukraine as Oleh. Oleh's son served as a Ukrainian church minister in Saskatoon. Over the years, our families got to know each other well.

### An Afterthought by the Authors: Significance of Oleh's Work to Humanity

To understand the impact of Dr. Oleh Hornykiewicz's contribution to chronic progressive neurological disease, we need to understand the status prior to his seminal discoveries. The clinical diagnosis of PD was not difficult to make for a neurologist. After the diagnosis, the neurologist would tell the patient that PD is a progressive disease for which there is no cure. The only treatment available was via anticholinergic drugs that had modest symptomatic benefit in some cases. By contrast, the effect of l‐dopa therapy was and remains “miraculous.” Oleh's initial observations led to oral l‐dopa as the standard treatment for PD. After more than 5 decades, it remains the best symptomatic drug treatment for PD. There are more than 7 million parkinsonian patients in the world at any time. Every patient with PD who can afford it is being treated with l‐dopa. If only 5 million patients were being treated at a given time, with significant improvement of their symptoms during a 50‐year interval, l‐dopa therapy would have improved 250 million person‐years of human life. There is no other drug for chronic progressive neurodegenerative diseases that can approach that impact. Even for other common diseases, there are not many drugs that remain the gold standard for such a long time.

### 14.2 Special Awards

For his extraordinary contributions, Oleh received both numerous and major awards such as the following: The Wolf Foundation Award in Medicine, American Parkinson's Disease Association Award, and Warren Alpert Foundation Prize. Most of us working in the PD field expected that some day Oleh Hornykiewicz would be recognized by the Nobel Prize committee for his outstanding contribution to the fields of medicine, neuroscience, and pharmacology. Inexplicably, he was overlooked for the 2000 Nobel Prize for Medicine or Physiology, which was awarded to 3 other scientists on the topic of PD and molecular neurosciences. A number of individuals wrote personal letters to different journals, and 275 scientists from around the world wrote an open letter to the Nobel Prize committee that had overseen the 2000 decision, indicating their disapproval for his omission.^21^ Consistent with the historical records, the outcome remained unchanged; however, Oleh was pleased that so many of his colleagues valued his work so much that they put their signature on this open letter.

Former colleagues, trainees, and fellow scientists around the globe are confident that Oleh's contribution to the medical sciences will remain a major milestone in the history of PD. To us, the collective body of work he produced remains the most significant contribution to the understanding and treatment of chronic brain disorders.

On behalf of millions of patients and scientists who have benefited from your discoveries, we collectively express, thank you, Oleh!

## Full financial disclosures for the previous 12 months

Dr. Ali Rajput has no disclosures. Dr. Stephen Kish has received funding from the US National Institutes of Health NIAAA 026680 and from an investigator‐sponsored grant from Jazz Pharmaceuticals, which are both unrelated to the research covered in this article.
